# Targeting lung cancer: synergistic therapeutic strategy of cuproptosis and immunogenic cell death

**DOI:** 10.3389/fcell.2026.1732351

**Published:** 2026-05-04

**Authors:** Yan Yang, Wanjuan Pei, Tao Ren, Yiru Wang, Ling Dai, Baierna Maimaitiaili, Bin Meng, Qun Tang, Huiling Feng

**Affiliations:** 1 Hunan University of Chinese Medicine, Changsha, China; 2 Yueyang County Health Bureau, Yueyang, China

**Keywords:** cuproptosis, immunogenic cell death, immunotherapy, lung cancer, molecular target

## Abstract

Cu, a common essential trace element in the human body, is involved in multiple physiological and pathological processes. Cuproptosis, a recently proposed form of cell death, is triggered by Cu overload. It primarily targets lipoylated proteins in the tricarboxylic acid cycle, induces proteotoxic stress, and impairs mitochondrial respiration. Cuproptosis can induce immunogenic cell death (ICD), which triggers adaptive immunity in response to cellular stress, thereby stimulating an immune response. Conversely, ICD may also induce cuproptosis, indicating an interaction between the two; this mechanism should provide insights in lung cancer treatment. Lung cancer is a common disease that endangers human health and is characterized by high mortality and incidence rates. Despite the availability of therapeutic regimens such as surgery, radiotherapy, chemotherapy, and immune checkpoint inhibitors, their prognosis remains controversial. This review innovatively proposes that the combination of these two forms of cell death, together with their interaction, may target biomarkers of various lung cancers. This study provides a theoretical basis for addressing the challenges encountered in lung cancer treatment.

## 1 Introduction

Approximately 2.2 million new lung cancer cases occur annually, accounting for 11.4% new cancer cases and ranking second worldwide in terms of incidence (GLOBOCAN 2020 global cancer statistics). However, lung cancer remains the leading cause of cancer-related deaths, with an estimated 1.8 million annual deaths (18%) (Sung et al., 2021). According to its main histological characteristics, lung cancer can be divided into non-small cell lung cancer (NSCLC), which accounts for approximately 85% cases, and small cell lung cancer (SCLC). NSCLC can be further classified into lung adenocarcinoma (LUAD), lung squamous cell carcinoma (LUSC), and large cell carcinoma. Early-stage NSCLC is often treated with surgery, whereas advanced-stage disease is treated with chemotherapy or radiotherapy (Li et al., 2023). SCLC, a lethal neuroendocrine tumor characterized by early widespread metastasis and high aggressiveness, is mostly accompanied by the inactivation of the tumor suppressor genes *RB1* and *TP53*, and more than 95% cases are associated with smoking as a risk factor. First-line treatment consists of combined platinum-based chemotherapy and radiotherapy; however, the prognosis remains poor after chemoradiotherapy (Wang Q. et al., 2023). Moreover, chemotherapeutic drugs exhibit inherent resistance, which poses significant challenges. Immunotherapy for lung cancer, including immune checkpoint inhibitors (already approved for lung cancer) and chimeric antigen receptor (CAR) T cells, is gradually emerging and shows promise as an adjuvant strategy. However, owing to rapid lung cancer progression, tumor microenvironment heterogeneity, drug resistance emergence, and recurrence (Xiao et al., 2025), whether such therapies can be used as first-line treatments require further extensive research.

Owing to the limitations of lung cancer therapy, cell death may provide targets for combination treatment. Cuproptosis is a ferredoxin 1 (FDX1)-mediated cell death mechanism characterized by Cu ion overload, which targets the tricarboxylic acid (TCA) cycle and induces proteotoxic stress (Kahlson and Dixon, 2022). The TCA cycle provides nutritional support for cancer cell survival and Cu ions are involved in regulating programmed death ligand-1 (PD-L1) expression. Therefore, cuproptosis combined with an anti-PD-L1 (αPD-L1) inhibitor exerts a sensitizing effect on immunotherapy of lung cancer (Xu Y. et al., 2025). Moreover, the combination of αPD-L1 and chemotherapy is often used in metastatic NSCLC; however, it fails to exert satisfactory synergistic effects, which may be attributed to the reduction in chemokines and corresponding CD8^+^ T cell loss (Limagne et al., 2022). As the effector cells of anti-tumor immunity, CD8^+^ T cells are frequently induced by immunogenic cell death (ICD). ICD is a regulated cell death process in which cells release damage-associated molecular patterns (DAMPs) and recruit dendritic cells (DCs) for antigen presentation, thereby activating cytotoxic cells and ultimately eliciting an adaptive immune response (Galluzzi et al., 2020). This process involves endoplasmic reticulum (ER) stress, autophagy, and cell death regulation. In studies of LUAD, induction of immunogenicity helps enhance the immunotherapeutic effect of αPD-L1, but its specific regulatory mechanism remains unexplored. However, studies on other types of lung cancers are limited (Zhang et al., 2025). Cuproptosis may interact with ICD, especially in cancers. Examples include inhibiting cuproptosis by ER stress-related regulators, ICD induction, and immune responses triggered by cuproptosis. These processes involve complex mechanisms requiring further investigation and validation.

This review discusses the potential targeting roles of cuproptosis, ICD, and their interaction in various types of lung cancer (LUAD, LUSC, and small cell lung cancer), aiming to provide theoretical support for the research on these two types of cell death in lung cancer. Meanwhile, we focus on the characteristic molecular changes in different types of lung cancer, explore the potential molecular targets of these two forms of cell death, provide directions for further research on lung cancer, and are expected to reveal new directions in lung cancer research.

## 2 Concept and mechanism of ICD

### 2.1 What is ICD?

ICD is a regulated cell death process that elicits an adaptive immune response under stress conditions and usually requires an environment with sufficient antigenicity and adjuvanticity (Galluzzi et al., 2020). In addition, such immunogenicity generally needs to manifest in a certain cytotoxic environment and microenvironment, where adjuvanticity is reflected by the release of DAMPs during cell death or stress—endogenous factors that trigger immune responses. Common types include DNA, nucleic acids, intracellular metabolites, lipids, and the extracellular matrix (Chen F. et al., 2024). When they appear as signals, they recruit DCs, a type of antigen-presenting cell, to take up antigens that are then presented to T lymphocytes to activate them. Cytotoxic T lymphocytes (CTLs) are the executors of the ICD process (Kroemer et al., 2022; Galluzzi et al., 2024). ICD can be regarded as a special form of cancer cell death that enhances anti-tumor immune surveillance. DAMPs are recognized by pattern recognition receptors (PRRs), thereby enhancing effector T cell infiltration (Yu S. et al., 2023). RIPK1 is located downstream of PRR molecules. Acute RIPK1 knockdown activates the Tumor Necrosis Factor Receptor 1 (TNFR1) and TLR3/4 pathways, thereby overactivating NF-κB, mitogen-activated protein kinase (MAPK), and IFN signaling; promoting ICD; and driving long-lasting anti-tumor immunity (Mannion et al., 2024). IFN acts as another DAMP. Studies have confirmed that inhibition of its negative regulator, ubiquitin-specific protease 18 (USP18), can induce cell death, which also induces ICD (Arim et al., 2023).

**FIGURE 1:**
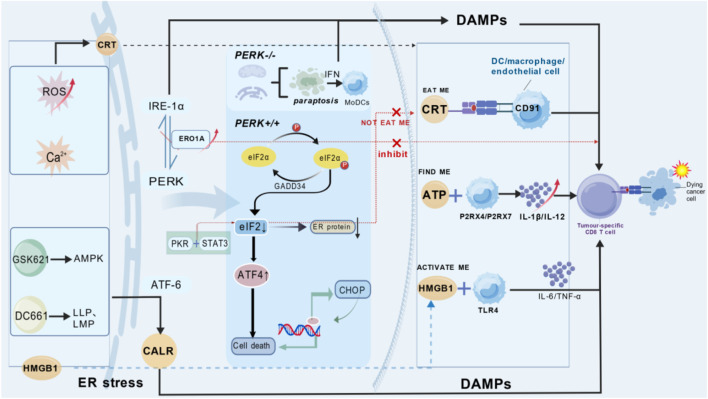
This schematic diagram systematically illustrates the core role of the “PERK-eIF2α-ATF4” axis-driven immunogenic cell death (ICD) signaling network in determining the fate of tumor cells. The core content of the figure includes: (1) ICD Triggering: Four types of exogenous or drug stimuli (radiotherapy/ROS↑, TRPML1→Ca^2+^ homeostasis imbalance, GSK621→AMPK↑, DC661→lysosomal membrane permeabilization LLP/LMP) converge into ER stress signals, setting the threshold for ICD. (2) PERK Main Pathway Execution: The PERK→p-eIF2α→ATF4→CHOP cascade amplifies the transcriptional program. It releases three types of DAMPs in chronological order: ① Early stage: CRT/CALR translocates to the cell membrane via vesicles (“eat me” signal); ② Middle stage: ATP is actively exported through pannexin channels (“find me” signal); ③ Late stage: HMGB1 is passively released (“active me” signal). (3) DC Recognition and T Cell Cross-Activation: It promotes the maturation of dendritic cells and activates CD8^+^ T cells, ultimately mediating the specific killing of tumor cells. (4) Bypass/Alternative Death: When PERK is absent, cells bypass classical apoptosis and switch to paraptosis (characterized by vacuolization of ER and mitochondria). They still release DAMPs, maintaining ICD output. (5) Negative Brakes: ① Increased ERO1A disrupts the balance between PERK and IRE1α, impairing the subsequent function of CD8^+^ T cells; ② Increased STAT3 blocks p-eIF2α and upregulates CD47 (“don’t-eat-me” signal), leading to immune evasion. The descriptions of key molecules and pathways are as follows: PERK, Protein kinase R-like endoplasmic reticulum kinase; eIF2α, Eukaryotic translation initiation factor 2α; ATF4, Activating transcription factor 4; CHOP, C/EBP homologous protein; HMGB1, High-mobility group box 1 protein; TRPML1, Transient receptor potential cation channel, mucolipin subfamily member 1; GSK621, Small-molecule agonist of AMPK; DC661, Lysosomal acidification inhibitor; LLP/LMP, Lysosomal membrane permeabilization; P2X7, Purinergic receptor P2X7; NLRP3, NOD-like receptor family pyrin domain containing 3; TLR4, Toll-like receptor 4; CD91, Low-density lipoprotein receptor-related protein 1; STAT3, Signal transducer and activator of transcription 3; ERO1A, Endoplasmic reticulum oxidoreductase 1α; CD47, Integrin-associated protein; THD, Small molecule targeting secretory autophagy. This figure was generated using the Biogdp platform.

Meanwhile, antigenicity can be manifested as follows: cells expressing a high antigen phenotype, such as malignant cells, infected cells, and dying cells, can be recognized by host naïve T cells and initiate adaptive immunity; healthy cells also undergo ICD via post-translational modifications. In contrast to traditional immune escape, Kacen et al. found that certain post-translationally modified peptides in cancer cells can be presented to the immune system by HLA to participate in the immune response (Kacen et al., 2023). During ICD, calreticulin (CRT) and heat shock proteins (HSP70 and HSP90) act as “eat-me” signals, interact with the CD91 receptor in phagocytes, and enable them to effectively phagocytose dead cells. Adenosine Triphosphate (ATP) released by dead cells serves as a “find-me” signal, facilitating DC and macrophage infiltration in tumor sites (Kar et al., 2023). Thus, DAMPs, such as high-mobility group box 1 (HMGB1), calreticulin (CRT), and ATP, are regarded as ICD occurrence markers and widely used in studies on ICD induction in cancer (Fan et al., 2024) (Figure 1).

### 2.2 ICD mechanism

ER stress induction triggers ICD. ER stress is initiated by three transmembrane sensors: PERK, IRE1, and activating transcription factor 6 (ATF6). ICD often occurs under intracellular Ca^2+^ homeostasis imbalance, or when other factors activate the PERK/eIF2α/ATF4/CHOP axis to induce ER stress, thereby promoting early CRT exposure, ATP release, and HMGB1 release, ultimately enhancing the immune response (Li et al., 2021). For example, the lysosomal calcium channel TRPML1 may mediate ER stress by regulating calcium levels, thereby inducing ICD (Zhu et al., 2023). Similarly, during the radiotherapy of tumor cells, high reactive oxygen species (ROS) levels translocate CRT from the ER membrane to the cell membrane or disrupt the cell membrane, resulting in the extracellular release of HMGB1 during late-stage apoptosis (Huang et al., 2021). HMGB1 is released from the nucleus in the vicinity of dying cells and stimulates the antigen-presenting function of DCs by binding to Toll-like receptor 4 (TLR4) on DCs. These DAMPs induce naïve DC maturation and promote immune responses (Ahmed and Tait, 2020). However, Mandula et al. found that tumor cells deficient in the ER stress-related kinase PERK undergo a form of cell death called paraptosis under ER stress. Paraptosis is characterized by the extensive vacuolization of the ER and mitochondria, which release DAMPs to promote ICD. Type I IFN drive the conversion of common monocyte precursors (cMoPs) to monocyte-derived DCs (MoDCs), which have strong immunostimulatory and phagocytic capacities and activate anti-tumor T cell responses (Zheng et al., 2023; Mandula et al., 2022). The small molecule GSK621 induces PERK-dependent ER stress by activating adenosine 5′-monophosphate-activated protein kinase (AMPK), ultimately leading to CALR exposure on the cell surface to enhance adjuvanticity. It also promotes bone marrow-derived DC activation and maturation in mouse leukemia cells and stimulates immune responses (Mondesir et al., 2023). In the tumor microenvironment (TME), ERO1A is upregulated during ER stress to protect cells, which also impairs the function of CD8^+^ T cells. Blocking ERO1A in tumor cells enhances protective T cell immunity, disrupts the signaling balance between IRE1α and PERK, triggers ER stress, and induces a lethal unfolded protein response during ER stress, ultimately triggering ICD to enhance anti-tumor immunity (Liu et al., 2023). The downstream of PERK is eIF2α, and eIF2α activation is necessary for CALR translocation in ICD. Studies on liver cancer have found that STAT3 can bind to the promoter of the “do not eat me” molecule CD47 and PKR to downregulate eIF2α activation. Therefore, STAT3 inhibition can induce ICD to enhance anti-tumor immunity (Li Y. et al., 2022). Previous studies have shown that ER stress induces autophagy. Low-level autophagy exerts a protective effect on tumor cells; meanwhile, autophagy degrades immunoproteasomes and antigen-presenting molecules via intact lysosomes, resulting in immune escape. In contrast, when proteasome membrane peroxidation is equivalent to labeling cancer cells (characterized by CALR expression), thereby regulating tumor immunity (Bhardwaj et al., 2023). The lysosomal inhibitor DC661 induces lysosomal lipid peroxidation (LLP) and lysosomal membrane permeabilization (LMP), leading to lysosomal cell death in tumor cells. This increases CALR expression, thereby enhancing T cell-mediated toxicity and inducing ICD (Phadatare and Debnath, 2023). In contrast, high levels of autophagy can induce tumor cell death, thereby releasing DAMPs to reshape the TME (Zhang S. et al., 2023). Moreover, ICD biomarkers rely on autophagosome formation. Moreover, secretory autophagy induced by thioxanthone (THD), which bypasses the lysosomal pathway, may occur independently of ER stress. Nevertheless, both processes promote DAMP release from dying cancer cells, thereby enhancing immune activation (Tran et al., 2023). This suggests that cell death type influences ICD efficacy. Further exploration of its relationship with other forms of cell death may provide novel breakthroughs in tumor immunity.

## 3 Molecular mechanism of cuproptosis

Cuproptosis is a novel mitochondrial metabolism-dependent programmed cell death mechanism triggered by excess Cu ions. Owing to its unique immunomodulatory potential, it has gradually become a research hotspot at the intersection of cell death and metabolism in recent years (Kahlson and Dixon, 2022). In tumor research, cuproptosis regulates metabolic reprogramming, oxidative stress levels, and the TME, providing a novel strategy for tumor therapy (Jiang et al., 2022; Zhang X. et al., 2023). Tsvetkov et al. first discovered that cuproptosis is distinct from other cell death forms (Tsvetkov et al., 2019). When mitochondrial metabolism changes, cellular oxidative phosphorylation is enhanced, while the affinity between Cu ionophore Elesclomol (ES) and Cu^2+^ increases. The ES–Cu^2+^ complex effectively delivers Cu to mitochondria under the action of FDX1 and promotes cell death by enhancing the oxidative stress response in tumor cells. Studies have found that the core site of cuproptosis occurrence is the mitochondrion, which is closely related to the energy metabolism process of mitochondria, among which the TCA cycle plays a key role in cuproptosis-induced cell death (Zhang Q. et al., 2022). When intracellular Cu^2+^ is overloaded, Cu^2+^ specifically targets and binds to a class of special proteins in the TCA cycle, namely lipoylated dihydrolipoamide S-acetyltransferase (DLAT), which undergo abnormal aggregation and oligomerization, forming insoluble protein aggregates, thereby disrupting the stability of Fe–S cluster proteins in the mitochondria and inducing cytotoxicity and cell death (Li et al., 2017). Zhang et al. proposed that this form of cell death is closely associated with the massive production of hydroxyl radicals by Cu complexes through the Fenton reaction, which leads to intense oxidative stress and DNA damage, thereby directly oxidizing and lysing the plasma membrane, as well as massive intracellular ROS generation (Zhang X. et al., 2023). Among these, p53 plays a key role (Ostrakhovitch and Cherian, 2005). Specifically, Cu can transactivate p53, upregulating Bcl-2, Bax, and other proteins. The related products selectively act on the mitochondria, increasing their permeability and ROS production, thereby promoting oxidative stress.

As a core regulatory factor in the cuproptosis pathway, FDX1 plays a key role in triggering the cuproptosis pathway (Liu X. et al., 2025; Song et al., 2025). On one hand, the lipoylation process of proteins such as DLAT requires FDX1 to provide the necessary S source to trigger ligation between the lysine residues of DLAT and lipoyl groups. On the other hand, FDX1 can also act as a reducing agent for Cu^2+^. By directly binding to ES, it inhibits Fe–S cluster biosynthesis, promotes Cu^+^ formation, and facilitates the binding process between Cu^+^ and lipoylated DLAT. Conversely, Cu^+^ formation can also upregulate mitochondrial metabolism and FDX1 enzymatic activity, further promoting cytotoxicity (Shi Z. et al., 2025). Lipoic acid synthase (LIAS) catalyzes nascent lipoic acid synthesis through its interaction with FDX1 and transfers lipoic acid to target proteins, such as DLAT, thereby completing the lipoylation process (Dreishpoon et al., 2023). Among them, lipoyltransferase 1 (LIPT1) plays an important “assembly” role and serves as a key bridge connecting lipoic acid metabolism and cuproptosis execution (Zhao et al., 2024). Therefore, inhibiting LIPT1 function renders cells resistant to cuproptosis. Studies have found that the above process is often dependent on an active mitochondrial respiration process (Tsvetkov et al., 2022). Proteins such as DLAT and dihydrolipoamide dehydrogenase (DLD) are extensively lipoylated by enzymes such as LIAS upon robust activation of the TCA cycle, thereby providing sufficient binding sites for intracellular overloaded Cu ions and inducing the oxidative stress process. As an important Cu transporter, SLC31A1/CTR1 maintains intracellular Cu homeostasis by transmembrane Cu^2+^ transport. Its expression level is directly associated with intracellular Cu^2+^ levels, thereby determining cell sensitivity to cuproptosis (Huo et al., 2023). In addition, deacetylation by sirtuin 7 (SIRT7) promotes FDX1 and SLC31A1 expression, leading to cuproptosis, mitochondrial dysfunction, and accelerates cell death. However, intracellular Cu^2+^ overload in turn reduces SIRT7 level (Kong et al., 2025). Under physiological conditions, ATP7A/ATP7B is the core transporter that maintains intracellular Cu homeostasis. ATP7B is localized in the liver and can export excess Cu out of cells, whereas ATP7A is located in the trans-Golgi network and mainly expressed in the small intestine, where it can transport Cu to specific sites of relevant intracellular enzymes. Both play the role of “balancers” in the cuproptosis mechanism (Guo et al., 2025). Dysregulation of their functions leads to an intracellular Cu imbalance and triggers cuproptosis. As important pyruvate dehydrogenase components, PDHA1/PDHB are mainly located in the mitochondrial matrix, cooperate with the energy production process of the TCA cycle, and are important targets for oligomerization and proteotoxicity during cuproptosis (Shi X. et al., 2025).

**FIGURE 2:**
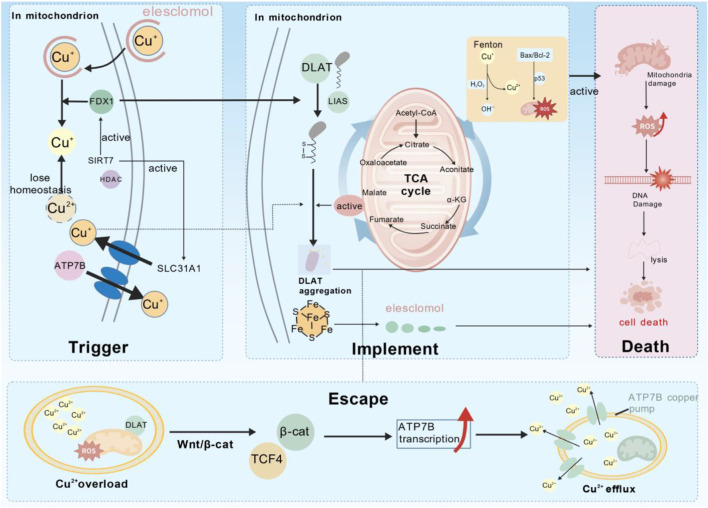
This schematic diagram systematically illustrates the fate decision process of the cuproptosis signaling axis in tumor cells, with a focus on dissecting how tumor cells trigger death programs via mitochondrial quality control (MQC) imbalance under copper overload stress, as well as their precise molecular pathways for evading cuproptosis. The core content of the diagram includes: (1) Cuproptosis Triggering, (2) Cuproptosis Execution (Death), and (3) Cuproptosis Outcome (Escape). ROS, hypoxia, and the mitochondrial dynamics protein DRP1 run through these three levels, providing novel targets for metabolism-immunity combination therapy. The descriptions of key molecules and pathways are as follows: FDX1, Ferredoxin 1; DLAT, Dihydrolipoamide S-Acetyltransferase; LIPT1, Lipoyltransferase 1; LIAS, Lipoic Acid Synthetase; ATP7B, Copper-Transporting P-Type ATPase; β-cat, β-catenin; TCF4, T-Cell Factor 4; SIRT7, Sirtuin 7; CTR1/SLC31A1, Copper Import Transporter; Cu^2+^, Copper Ion (Divalent); Cu^+^, Copper Ion (Monovalent); Fe-S, Iron-Sulfur Cluster; ROS, Reactive Oxygen Species; Bax/Bcl-2: Apoptosis Regulators. This figure was generated using the Biogdp platform.

In addition, when applying Cu homeostasis in clinical therapy, attention should be paid to the high-dimensional framework of systemic Cu homeostasis. As mentioned earlier, under physiological conditions, the body maintains a low serum-free Cu concentration through various proteins such as ATP7A/ATP7B, SLC31A1/CTR1, and Cu chaperone antioxidant protein 1 (ATOX1), thereby reducing the Fenton reaction, oxidative stress, and non-target tissue accumulation mediated by it (Guo et al., 2025). Recent studies have found that under Cu^2+^ overload, cancer cells activate the Wnt/β-catenin signaling pathway, promote the binding of the β-catenin/TCF4 complex to ATP7B, leading to upregulated ATP7B expression, increased Cu efflux, and decreased cytoplasmic Cu concentration, thereby enabling tumor cells to escape the cuproptosis process (Liu et al., 2024). This provides a new strategy for cancer therapy and suggests that simple systemic Cu supplementation has a very limited effect on changing the local effective Cu concentration in tumors and may even induce Cu accumulation toxicity in organs such as the liver, nerves, and kidneys owing to the compensatory efflux of normal tissues (Tsvetkov et al., 2022). Further studies have found that Cu overload can also inactivate mitochondrial Fe–S cluster proteins through an FDX1-independent pathway (Lu et al., 2025). This finding indicates that the toxic damage of cuproptosis is not only limited to lipoylated TCA cycle proteins, but also involves the transmission of the electron respiratory chain, providing a broad insight for clinical development of cuproptosis inducers. Therefore, the clinical application of cuproptosis strategies needs to be carried out on the premise of establishing a “therapeutic window,” that is, the Cu ion concentration at the tumor site is below the upper limit of FDX1 reduction capacity, while the circulating Cu level is controlled within the compensatory capacity of ATP7B/ATOX1, to ensure the sufficient aggregation of lipoylated proteins and reduce the toxic accumulation in non-target tissues. Lu et al. have preliminarily verified the feasibility of this strategy in tumor cuproptosis therapy by utilizing the TME-responsive release property of nanoparticles to spatiotemporally control Cu ion release, which can effectively trigger tumor cuproptosis and ensure that the systemic Cu exposure level is within a safe range (Mao et al., 2025) (Figure 2).

## 4 Cross-mechanisms and synergistic effects between ICD and cuproptosis

### 4.1 Interactions between ICD and cuproptosis

**FIGURE 3:**
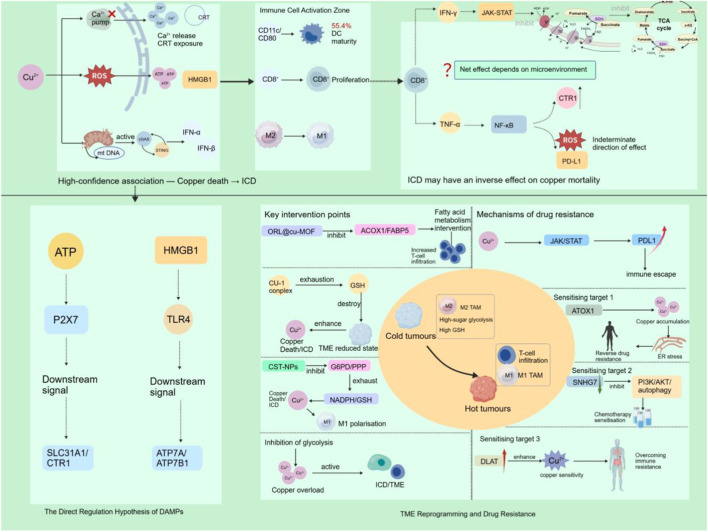
Evidence-stratified interplay between cuproptosis and ICD. Solid arrows: validated pathways; dashed arrows: indirect evidence; dashed arrows with question marks: speculative associations.Copper overload triggers ICD via ER stress, oxidative stress, and mitochondrial stress. In murine models, cuproptosis-inducing nanomedicines promote DC maturation, CD8^+^ T cell proliferation, and M1 polarization. Radiotherapy doubles DC maturation; adding PD-1/PD-L1 blockade achieves 60% 50-day survival and complete metastasis suppression. ICD-activated IFN-γ and TNF-α remodel metabolism: IFN-γ suppresses mitochondrial respiration, potentially reducing cuproptosis sensitivity; TNF-α modulates CTR1 expression time-dependently. Net effects are context-dependent. Early DAMPs may regulate copper transporters via P2X7/TLR4-NF-κB—a hypothesis requiring validation. Cuproptosis and ICD reprogram TME through GSH depletion, lipid remodeling, and glycolysis inhibition, converting “cold” to “hot” tumors. CST-NPs elevate M1/M2 ratio from 0.8 to 3.4 with 88.3% tumor inhibition. Clinically, DLAT overexpression correlates with ICB resistance in NSCLC. Targeting ATOX1, SNHG7, or DLAT sensitizes to chemotherapy and reverses immune resistance. DAMPs, Damage-associated molecular patterns; HMGB1, High mobility group box 1; cGAS, Cyclic GMP-AMP synthase; STING, Stimulator of interferon genes; JAK-STAT, Janus kinase-signal transducer and activator of transcription; NF-κB, Nuclear factor kappa-light-chain-enhancer of activated B cells; CTR1, Copper transporter 1; SDH, Succinate dehydrogenase; FH, Fumarate hydratase; IDH, Isocitrate dehydrogenase; P2X7, P2X purinoceptor 7; SLC31A1, Solute carrier family 31 member 1; ATP7A/ATP7B, ATPase copper transporting alpha/beta; ORL@cu-MOF, Orlistat-loaded copper-based metal-organic framework; ACOX1, Acyl-CoA oxidase 1; FABP5, Fatty acid binding protein 5; CU-1 complex, Copper complex CU-1; GSH, Glutathione; CST-NPs, Copper silicate nanoparticles; ATOX1, Antioxidant 1 copper chaperone; SNHG7, Small nucleolar RNA host gene 7; PI3K/AKT, Phosphatidylinositol 3-kinase/protein kinase B; DLAT, Dihydrolipoamide S-acetyltransferase.

ICD and cuproptosis are two distinct forms of regulated cell death. They exhibit substantial molecular crosstalk in triggering stress responses, signal transduction, and anti-tumor immunity, and have recently emerged as novel hotspots in anti-cancer therapy; however, the evidence for their interaction is asymmetric. Based on the current studies, these relationships can be categorized into three confidence levels to clearly distinguish between validated, indirectly supported, and speculative associations (Figure 3).

#### 4.1.1 High-confidence associations

Cuproptosis exerts Cu-dependent ICD effects, which can activate cellular immune responses and subsequently trigger extensive intracellular stress (Zhou et al., 2025). This activates the canonical DAMP release pathway of ICD, converting metal-dependent cytotoxicity into danger signals recognizable by the immune system and eliciting anti-tumor immune responses, thereby significantly suppressing tumor growth. ER stress and Ca^2+^ release are key bridges connecting ICD and cuproptosis (Li J. et al., 2025). Intracellular Cu overload induces intense proteotoxic and oxidative stress, leading to massive ATP consumption. The ER is rapidly affected and undergoes stress, severely impairing the calcium pump function. This further disrupts intracellular calcium homeostasis, translocating CRT to the cell membrane surface and emitting an “eat-me” signal, which triggers ICD. Consequently, it promotes the tumor infiltration of CTLs and enhances the recognition and phagocytosis of dead tumor cell debris by immune cells through the antigen presentation effect. Furthermore, the oxidative stress process triggered by cuproptosis promotes ATP and HMGB1 release (Li Y. et al., 2024). ATP itself exerts a recruitment effect, whereas HMGB1 can bind to TLR4 on the surface of DCs, thereby facilitating tumor cell localization by immune cells and enhancing host anti-tumor immune response (Fang et al., 2014).

This causal chain was further validated by multiple experiments. Luo et al. demonstrated via *in vitro* and *in vivo* experiments that the ES–Cu-based nanomodulator, which induces cuproptosis, elevated the levels of CARL, HMGB1, and ATP *in vitro*, triggered ICD, and was accompanied by 55.4% DC maturation and a 3.2-fold increase in CD8^+^ T-cell proliferation (Luo et al., 2024). Furthermore, compared with other preparations, this nanomodulator significantly suppressed tumor growth and prolonged survival in mice, indicating a robust anti-tumor immune effect. Cuproptosis elicits ICD mainly through danger signal release from cuproptotic tumor cells. These signals upregulate CD11c and CD80 expression, effectively activate the function of antigen-presenting cells (APCs), and promote the polarization of macrophages toward the anti-tumor M1 phenotype, thereby initiating the adaptive immune response (Li J. et al., 2025). Based on this characteristic, researchers have proposed through *in vitro* and *in vivo* experiments that compared with tumor suppression by radiotherapy (RT) alone, inducing Cu-triggered hydroxyl radical generation and GSH depletion while synergizing with RT to inhibit tumor volume not only induces ICD, but also doubles the maturation proportion of DCs (Wang et al., 2021). Cuproptosis can trigger mitochondrial stress responses, resulting in mitochondrial DNA (mtDNA) leakage, increased cytoplasmic mtDNA content, and cyclic GMP–AMP synthase (cGAS) recognition. This further activates the cGAS–STING pathway, producing IFN-α/β and various inflammatory factors, which in turn promotes DC maturation and enhances the ability of CD8^+^ T cells to kill tumor cells (Tao et al., 2025; Jiang et al., 2025). Liu et al. observed that the increase in hydroxyl radicals triggered by Cu^+^ can also induce an ICD response in mouse breast cancer (4T1) cells through this pathway. Therefore, long-term *in vivo* experimental results indicate that Cu-containing nanoparticles exhibit low toxicity, and when combined with PD-1/PD-L1 therapy, mice show no intrapulmonary tumor metastasis, with 60% survival rate at 50 days. This finding provides additional possibilities for tumor immunotherapy based on the combination of cuproptosis and ICD (Liu B. et al., 2025). These studies indicated that cuproptosis can act as an upstream signal to effectively promote ICD in multiple ways. Its essence lies in converting cell death induced by Cu metabolism disorders into “danger signals” that activate the body’s anti-tumor immune response. Therefore, intervention in the specific pathways underlying cuproptosis may represent a novel direction for tumor immunotherapy.

#### 4.1.2 Indirect evidence

Whether ICD can influence cuproptosis progression is not known clearly. Specifically, direct evidence of such a reverse regulatory relationship is currently lacking; however, some evidence suggests that the remodeling of the immune microenvironment activated by ICD may indirectly enhance the sensitivity of tumor cells to cuproptosis. *In vivo* studies have shown that primary tumors are eliminated during anti-tumor immune responses and untreated tumor cells can be inhibited through the “abscopal effect” (Wu et al., 2025). This indicates that ICD may indirectly promote additional tumor cell death processes, including the occurrence of cuproptotic effects. In addition, CD8^+^ T cells activated by ICD secrete large amounts of IFN-γ and TNF-α, which can remodel tumor cell metabolism. IFN-γ inhibits the activity of the mitochondrial respiratory chain in tumor cells through the JAK–STAT pathway (Zhang S. et al., 2024). Since cuproptosis is dependent on an active TCA cycle (Tsvetkov et al., 2022), IFN-γ can theoretically indirectly reduce the sensitivity to cuproptosis. In contrast, TNF-α may increase Cu transporter expression through the NF-κB pathway, and increased ROS and PD-L1 expressions may exert effects in different directions (Liu et al., 2024). Therefore, the direction of the net effect under these two conditions depends on various factors, such as the relative concentrations and metabolic levels of tumor cells, which urgently require support from definitive biochemical test data.

#### 4.1.3 Putative association

ATP and HMGB1 released during early ICD can activate the P2X7 and TLR4 receptors (Paredes et al., 2018). Although direct evidence for their involvement in SLC31A1/CTR1 and ATP7A/ATP7B expression regulation is lacking, they may theoretically participate in this process via the downstream NF-κB pathway, thereby modulating the sensitivity to cuproptosis. However, direct molecular mechanisms underlying ICD-induced cuproptosis, such as DAMP-promoted Cu influx, still require verification through mechanistic studies. Temporal analysis, genetic ablation, and single-cell multi-omics tracing can serve as key experiments to validate this hypothesis in the future. In addition, Deng et al. designed a nanoparticle with a T cell membrane coating (Deng et al., 2025), *In vitro*, it targets and localizes to tumor cells, induces a strong ICD effect, activates anti-tumor immune responses, and synergizes with photothermal therapy (PTT) and cuproptosis to efficiently eliminate tumor cells. Although this study did not directly provide evidence that ICD promotes cuproptosis, it simultaneously initiated two powerful cell death pathways through an exogenous photothermal signal and enabled their mutual promotion. This suggests that the synergistic effect of ICD and cuproptosis holds great potential for developing tumor therapy.

### 4.2 TME reprogramming

Notably, cuproptosis can not only directly induce tumor cell death, but also synergize with ICD to remodel the TME, which may be closely associated with the conversion of “cold tumors” to “hot tumors” (Huang et al., 2022). The TME provides tumor cells with sufficient energy and antioxidants to maintain their growth requirements and homeostasis. Impairment of immune cells in the TME leads to further tumor cell growth and spread (Cao et al., 2025). In recent years, tumor immunotherapy has gradually emerged and ICD plays a crucial role in promoting the immunogenic death of tumor cells (Peng et al., 2024). However, owing to the suppressive TME and insufficient immune system activation, immunotherapy still has many limitations in clinical applications. Therefore, inducing cellular cuproptosis to promote DAMP release and ICD, while disrupting the TME and converting suppressive “cold tumors” into immune cell-active “hot tumors,” thereby impairing tumor cell viability, has become a new direction for intervening in cancer progression (Wang et al., 2024a). Yun et al. (2025) encapsulated ES and Cu^2+^ into nanoparticles to design a TME-responsive nanoinducer, which can be released upon specific exposure to the TME, trigger cuproptosis, and detect DAMP release and function to confirm the occurrence of immune responses, exerting significant toxicity to *in vitro* cells, Meanwhile, using a mouse model showed that it promoted T cell activation and proliferation. Ultimately, this indicates that the suppressive TME was remodeled, resulting in 88.3% tumor inhibition rate and thereby extending the survival period of the mice. Glutathione (GSH) is an important molecule widely expressed in tumor cells and can exert an antioxidant effect by chelating Cu^2+^, thereby maintaining the reduced state of cells and creating an environment for the correct folding and solubility of proteins to counteract cytotoxicity (Encinas-Gimenez et al., 2025). Therefore, when GSH is depleted, the stability of the TME is disrupted, leading to the binding of Cu to misfolded proteins and accelerating the cell death process. Huang et al. found that using a Cu complex CU-1 can deplete intracellular GSH and induce hydroxyl radical production, thereby disrupting cellular redox homeostasis and triggering an apoptosis-independent ICD response (Huang et al., 2024). It significantly inhibits tumor cell growth and is low toxic. Cuproptosis and ICD can upregulate PD-L1 expression in tumor cells. Under the induction of cuproptosis, PD-1/PD-L1 inhibitors can effectively exert anti-cancer effects (Voli et al., 2020), which is closely associated with changes in the TME. The combined application of PD-1/PD-L1 inhibitors and cuproptosis can increase CTL infiltration and DC maturation, while reducing regulatory T cells (Tregs) in the TME. Clinical controlled studies have also found that the objective response rate (ORR) of PD-L1 inhibitors combined with anti-cancer drugs is generally increased, and this combination may induce ICD. Therefore, comprehensive immunotherapy combined with TME reprogramming can combat drug-resistant malignant tumors (Ji et al., 2025; Inoue and Narukawa, 2022).

Lipid metabolic reprogramming is an important reason for tumor cells to resist ICD and is key to fatty acid metabolism (Luo et al., 2023) Studies have shown that the metal–organic framework nanoparticle ORL@Cu–MOF can selectively localize to tumor cells in model mice and promote Cu^2+^ release and delivery, thereby inducing processes such as mitochondrial ROS production and ER stress (Li Z. Z. et al., 2025). *In vitro* studies have confirmed that ORL@Cu–MOF can also inhibit lipid metabolism-related enzymes such as acyl-CoA oxidase 1 (ACOX1) and fatty acid-binding protein 5 (FABP5) and interfere with the fatty acid metabolism process, thereby improving CD4^+^/CD8^+^ T cell infiltration in the TME and converting “cold tumors” to “hot tumors,” effectively improving tumor cell resistance to immunity and making up for the deficiencies of ES–Cu^2+^ in anti-cancer therapy (Guo et al., 2023). Tian et al., after comprehensively studying clinical patients with liver diseases, human hepatic stellate cells (HSCs), and animal models of liver fibrosis, found that the RAB18 protein involved in lipid metabolism can undergo liquid–liquid phase separation (LLPS), encapsulate lipid droplets to inhibit lipophagy, and disrupt the lipid metabolic balance of cells (Tian et al., 2025). This process is accompanied by the recruitment of carnitine palmitoyltransferase 1 (CPT1), whose activation promotes DLD succinylation, thereby converting the lipid metabolism arrest signal into a cuproptosis-triggering signal. This further indicates a potential close association between lipid metabolism and cuproptosis. The glycolytic activity of cancer cells is another important factor that enhances the suppressive state of the TME. By studying mouse squamous cell carcinoma, Li et al. found that disrupting cellular glycolysis can promote intracellular Cu overload, convert the TME from a suppressive state to an active state, and enhance the ICD induced by cuproptosis, thereby inhibiting tumor growth and distant metastasis, which was achieved using a nano-platform in animal models (Li Q. et al., 2025). The suppressive state of TME depends on the pro-tumor factors secreted by M2-type tumor-associated macrophages (M2-TAMs) and their role in impeding antigen presentation. To address this, a new material, CST–NPs, has been developed (Wang et al., 2026), *In vitro* and *in vivo* models have confirmed that it can inhibit glucose-6-phosphate dehydrogenase (G6PD) to block the pentose phosphate pathway (PPP), deplete nicotinamide adenine dinucleotide phosphate (NADPH) and GSH, increase intracellular Cu content, induce redox imbalance, and trigger ICD, thereby promoting the differentiation of TAMs into the M1 type. This suggests that cuproptosis and ICD synergy may bring hope for solving the problem of immune escape in tumors. Chen et al. found that bimetallic nanozymes can induce acylated protein and lipid peroxide aggregation, induce both ferroptosis and cuproptosis simultaneously, and activate immune responses by releasing danger signals such as HMGB1 and ATP, thereby remodeling the TME, efficiently disrupting the tumor defense system, and promoting tumor cell death (Chen et al., 2025). This suggests that the metal immune system has guiding significance in the research and development of anti-cancer drugs, and the potential therapeutic possibilities of other metal ions urgently need further exploration.

### 4.3 Immune resistance

Cu plays a dual role in tumor cells. On one hand, cuproptosis can induce ICD in tumor cells, thereby accelerating cancer cell death (Wang X. et al., 2023). However, since the interaction between PD-1 and its ligand PD-L1 is the core mechanism of tumor immune escape, Cu can upregulate PD-L1 expression in cancer cells by activating the JAK/STAT signaling pathway and promoting STAT3 and EGFR phosphorylation, thereby promoting tumor growth and immune escape (Qin et al., 2023). Therefore, disrupting Cu ion homeostasis can alter the immune escape ability of tumor cells, thereby enhancing their sensitivity to anti-cancer drugs. The liver is the main organ for Cu storage and excretion (Kirsipuu et al., 2020). ATOX1 distributes Cu from the liver to other tissues and organs via blood circulation. Studies have shown that inhibiting ATOX1 expression can promote the accumulation of chemotherapeutic drugs and Cu in tumor cells, achieve a chemosensitization effect, enhance ER stress and ICD effects, restore the immunogenicity of tumor cells, and provide a new solution for the resistance encountered in anti-cancer therapy (Ding et al., 2022; Hu et al., 2023). Wen et al. (2023) found that treating *in vitro* and *in vivo* tumor models with ES–CuCl_2_ can increase the expression levels of DLAT and HSP70 and promote the phosphorylation of mammalian target of rapamycin (mTOR), thereby reversing its inhibitory effect on autophagy and improving tumor drug resistance.

Numerous studies have found that cuproptosis has great potential to promote immune responses in lung cancer and improve the resistance of tumor cells to clinical anti-cancer drugs. Zhang K. et al. (2022) found that there is high expression of SNHG7 in docetaxel (DTX)-resistant tumor cells. Downregulating SNHG7 expression can reduce the IC_50_ of lung cancer cells to DTX and inhibit the proliferation and metastasis of cancer cells. From a molecular mechanism perspective, on one hand, SNHG7 exerts a positive feedback effect on the recruitment of Hu antigen R (HUR) and the expression of autophagy-related genes ATG5/ATG12, thereby promoting autophagy (Yu Z. et al., 2023). In contrast, SNHG7 adsorbs miR-543/miR-34a (She et al., 2023) to relieve its inhibition of phosphatidylinositol-4,5-bisphosphate 3-kinase catalytic subunit alpha (PIK3CA), thereby activating the PI3K/AKT signaling pathway, increasing the concentration of Cullin 4A (CUL4A) and the activity of ubiquitin ligase in macrophages, which facilitates M2 polarization. These are important reasons for the resistance of lung cancer cells to DTX; therefore, targeting SNHG7 can increase the sensitivity of lung cancer cells to anti-cancer drugs. DLAT is upregulated in both LUAD and LUSC. As one of the core targets of cuproptosis, the high DLAT expression implies that cancer cells may be more sensitive to Cu-induced oxidative stress, which is more likely to trigger the cuproptosis pathway and of great significance for reversing the resistance of tumor cells to immune checkpoint inhibitors (Yang et al., 2023). As mentioned earlier, cancer cells can escape the cuproptosis process by activating the MELK–PI3K/mTOR and Wnt/β-catenin signaling pathways, which is an important reason for the development of drug resistance in cancer cells. Therefore, targeting signaling pathways such as MELK–PI3K/mTOR and Wnt/β-catenin, synchronously attacking multiple related targets through horizontal combination, and combining with traditional radiotherapy, chemotherapy, and immunotherapy are expected to provide a more effective therapeutic method for such highly malignant and drug-resistant tumor cells (Liu et al., 2024; Yalimaimaiti et al., 2023) (Table 1).

**TABLE 1:** Cross-talk mechanisms and synergistic effects between ICD and cuproptosis.

Direction		Specific mechanisms	Function	Author	References
Interaction between ICD and Cuproptosis	High-confidence Association	Copper overload induces cuproptosis in cells, triggering proteotoxic stress and oxidative stress, activating the intracellular release pathway of damage-associated molecular patterns (DAMPs), and converting metal toxicity into recognizable danger signals for the immune system	Initiates anti-tumor immune responses and significantly suppresses tumor growth	Zhou Y. et al. (2025)	Zhou et al. (2025)
		Copper overload induces endoplasmic reticulum (ER) stress and calcium pump dysfunction, leading to intracellular calcium homeostasis imbalance, translocation of calreticulin (CRT) to the cell membrane surface, and emission of the “eat me” signal. It upregulates the expression of CD11c and CD80, activates antigen-presenting cells (APCs), and promotes the polarization of macrophages toward the M1 phenotype	Triggering immunogenic cell death (ICD) promotes the infiltration of cytotoxic T lymphocytes (CTLs) and antigen presentation, thereby enhancing the recognition and clearance of tumor debris by immune cells	Li J. et al. (2025)	Li J. et al. (2025)
		During cuproptosis, oxidative stress is enhanced, promoting the release of ATP and high-mobility group box 1 protein (HMGB1). HMGB1 binds to Toll-like receptor 4 (TLR4) expressed on the surface of dendritic cells	Recruits immune cells, enhances the recognition and attack of tumor cells, and elevates the anti-tumor immune response	Li Y. et al. (2023), Fang H. et al. (2014)	Li et al. (2023), Fang et al. (2014)
		The ES-Cu nanomodulator induces an increase in mitochondrial reactive oxygen species (ROS) levels, accompanied by the activation of immunogenic cell death (ICD)-related signals	It inhibits tumor growth and prolongs the survival time in mice	Luo Y. et al. (2024)	Luo et al. (2024)
​	​	Copper ions (Cu^+^ and Cu^2+^) induce hydroxyl radical generation and glutathione depletion, respectively, and act synergistically with radiotherapy to enhance the intracellular stress response	It increases the activation level of dendritic cells and enhances the immunogenic cell death (ICD) process	Wang Y. et al. (2021)	Wang et al. (2021)
		Cuproptosis induces mitochondrial stress; Cu^+^-mediated increase in hydroxyl radicals leads to the leakage of mitochondrial DNA (mtDNA) into the cytoplasm, which binds to cyclic GMP-AMP synthase (cGAS), activates the cGAS-STING pathway, and induces the expression of IFN-α/β and inflammatory factors	It promotes the maturation of dendritic cells and enhances the cytotoxic capacity of CD8^+^ T cells against tumor cells	Tao J. et al. (2025), Jiang Y. et al. (2025), Liu B. et al. (2025)	Tao et al. (2025), Jiang et al. (2025)
	Indirect Evidence	The anti-tumor immune response eliminates tumor cells outside the primary lesion via the abscopal effect, suggesting that ICD may indirectly promote the death of other tumor cells, although the underlying mechanism remains unclear	It provides a potential research direction for the synergistic anti-tumor effect of ICD and cuproptosis	Wu H. et al. (2025)	Wu et al. (2025)
		CD8^+^ T cells activated by ICD secrete IFN-γ and TNF-α, which remodel tumor cell metabolism; IFN-γ inhibits mitochondrial respiratory chain activity through the JAK-STAT pathway (cuproptosis is dependent on an active tricarboxylic acid (TCA) cycle, which theoretically reduces cuproptosis sensitivity); TNF-α may increase the expression of copper transporters, as well as the expression of ROS and PD-L1, via the NF-κB pathway	The net effect depends on the concentration and metabolic level, which requires further verification	Tsvetkov P. et al. (2019), Liu Y. T. et al. (2024), Zhang S. et al. (2024)	Tsvetkov et al. (2019), Liu et al. (2024), Zhang S. et al. (2024)
	Putative Association	ATP and HMGB1 released by ICD activate P2X7 and TLR4 receptors, which may affect the expression of copper transporters through the NF-κB pathway	Theoretically, it may affect cuproptosis sensitivity, and the direct mechanism remains to be verified	Paredes C. et al. (2018)	Paredes et al. (2018)
		T cell membrane-camouflaged nanoparticles induce immunogenic cell death (ICD) and activate immune responses, synergizing with photothermal therapy (PTT) and cuproptosis	It suggests the potential development space for the synergistic therapy of ICD and cuproptosis	Deng W. et al. (2025)	Deng et al. (2025)
Reprogramming of the tumor microenvironment		Multifunctional cascade bioreactors synergistically enhance immunogenic cell death (ICD) and the polarization of tumor-associated macrophages (TAMs), optimizing cancer immunotherapy	It reprograms M2-type tumor-associated macrophages (M2-TAMs) into an anti-tumor phenotype, overcoming the immunosuppressive tumor microenvironment (ITME)	Huang C. et al. (2022)	Huang et al. (2022)
		The tumor microenvironment (TME) provides energy and antioxidants for tumor cells to maintain their homeostasis; once the infiltration of immune cells is impaired, tumors will accelerate progression	This explains why disrupting the TME is required to restore immune surveillance	Cao Y. et al. (2025)	Cao et al. (2025)
		ICD plays a key role in enhancing immunogenic death of tumor cells and serves as a core component of immunotherapy	It provides a theoretical basis for enhancing immunotherapeutic efficacy in combination with cuproptosis	Peng Y. et al. (2024)	Peng et al. (2024)
		Inducing cuproptosis can promote the release of damage-associated molecular patterns (DAMPs) and trigger immunogenic cell death (ICD), while disrupting the suppressive tumor microenvironment (TME) and converting “cold tumors” into “hot tumors”	It impairs tumor viability and becomes a new strategy for cancer intervention	Wang Y. et al. (2024)	Wang Y. et al. (2024)
		Tumor microenvironment (TME)-responsive nanoparticles were constructed to specifically release ES and Cu^2+^ in the microenvironment, triggering cuproptosis and detecting the release of damage-associated molecular patterns (DAMPs) as well as immune cell infiltration	It was confirmed that cuproptosis can remodel the tumor microenvironment (TME) and prolong the survival time of mice	Yun K. et al. (2025)	Yun et al. (2025)
		Tumor cells highly express GSH, which chelates Cu^2+^ to maintain a reduced state, assist in proper protein folding, and counteract cytotoxicity	These findings indicate that GSH depletion can exacerbate cuproptosis and destabilize the TME.	Encinas-Gimenez M. et al. (2025)	Encinas-Gimenez et al. (2025)
​		The copper complex CU-1 was used to generate hydroxyl radicals and deplete GSH simultaneously, thereby disrupting redox homeostasis and inducing non-apoptotic ICD.	Restore homeostasis and induce non-apoptotic ICD, thereby achieving low-toxicity and high-efficiency anti-tumor effects and further disrupting the protective tumor microenvironment	Huang K. B. et al. (2024)	Huang et al. (2024)
		Cuproptosis and ICD upregulate PD-L1 expression in tumor cells.Combination with PD-1/PD-L1 inhibitors increases CTL infiltration and DC maturation, and reduces regulatory T cells	Synergism between immune checkpoint blockade and TME reprogramming overcomes drug resistance	Voli F. et al. (2020), Ji A. et al. (2025)	Voli et al. (2020), Ji et al. (2025)
		ORL@Cu-MOF selectively accumulates in tumors, promotes Cu^2+^ release, induces mitochondrial ROS and endoplasmic reticulum stress, and corrects abnormal lipid metabolism	It increases CD4^+^/CD8^+^ T cell infiltration, achieves “cold-hot” tumor conversion, and compensates for the deficiencies of ES-Cu^2+^ therapy	Luo M. et al. (2023), Li Z. Z. et al. (2025), Guo B. et al. (2023)	Luo et al. (2023), Li Z. Z. et al. (2025), Guo et al. (2023)
		After treatment of cells with garlic extract DAT, the RAB18 protein undergoes liquid–liquid phase separation and encapsulates lipid droplets to inhibit lipophagy, thereby disturbing lipid metabolic homeostasis. This process is accompanied by the recruitment of CPT1, activates the succinylation of DLD, and converts the lipid metabolism arrest signal into a cuproptosis-triggering signal	This reveals a close link between lipid metabolism and cuproptosis, providing a novel target for metabolism–cuproptosis combined therapy	Tian H. et al. (2025)	Tian et al. (2025)
		Treatment with the dual-reflective protease inhibitor Dream blocks glycolysis, induces intracellular copper overload, and relieves immunosuppression in the TME.	It enhances cuproptosis-induced immunogenic cell death and suppresses tumor growth and metastasis	Li Q. et al. (2025)	Li Q. et al. (2025)
​		CST-NPs inhibit G6PD, block the pentose phosphate pathway, deplete NADPH and GSH, elevate cytoplasmic copper levels, and initiate an immune response	It simultaneously resolves redox imbalance and immune escape, thereby remodeling the TME.	Wang Z. et al. (2026)	Wang et al. (2026)
		Bimetallic nanozymes promote the accumulation of lipoylated proteins and lipid peroxides, simultaneously induce ferroptosis and cuproptosis, and release danger signals such as HMGB1 and ATP.	It activates the immune response, remodels the TME, and efficiently disrupts the tumor defense system	Chen T. et al. (2025)	Chen et al. (2025)
Immune Resistance		Cuproptosis induces immunogenic cell death in tumor cells	It accelerates cancer cell death and weakens immune escape	Wang X. et al. (2023)	Wang X. et al. (2023)
		Copper upregulates PD-L1 expression by activating the JAK/STAT pathway and promotes the phosphorylation of STAT3 and EGFR.	It enhances tumor immune escape and promotes the development of drug resistance	Qin Y. et al. (2023)	Qin et al. (2023)
		Inhibition of the copper chaperone protein **ATOX1** reduces copper efflux, leading to the accumulation of hepatic copper in tumors, increasing endoplasmic reticulum stress and ICD.	It improves the sensitivity of chemotherapeutic drugs and reverses immune resistance	Kirsipuu T. et al. (2020), Ding F. et al. (2022), Hu X. et al. (2023)	Kirsipuu et al. (2020), Ding et al. (2022), Hu et al. (2023)
		In the ES-CuCl_2_-treated model, the expression of DLAT and HSP70 was upregulated, mTOR phosphorylation was restored, and the inhibition of autophagy was relieved.	It improves the drug-resistant microenvironment and enhances the response to subsequent therapy	Wen H. et al. (2023)	Wen et al. (2023)
		Downregulation of lncRNA SNHG7 reduces HUR recruitment and ATG5/ATG12 expression, thereby inhibiting autophagy. Meanwhile, it abolishes the sponging effect of SNHG7 on miR-543/miR-34a, reduces PI3K/AKT activity, and decreases M2 polarization	It decreases the IC50 of docetaxel and restores drug sensitivity in lung cancer cells	Zhang K. et al. (2022), Yu Z. et al. (2023), She K. et al. (2023)	Zhang K. et al. (2022), Yu Z. et al. (2023), She et al. (2023)
​		DLAT is upregulated in lung adenocarcinoma and squamous cell carcinoma, rendering cancer cells more susceptible to copper-induced oxidative stress and more prone to cuproptosis	It provides an actionable target for overcoming immune checkpoint inhibitor resistance	Yang Q. et al. (2023)	Yang et al. (2023)
		Cancer cells evade cuproptosis by activating the MELK-PI3K/mTOR and Wnt/β-catenin pathways, leading to drug resistance	Combined inhibition of these pathways can synergize with radiotherapy, chemotherapy, and immunotherapy to overcome highly resistant tumors	Liu Y. T. et al. (2024), Yalimaimaiti S. et al. (2023)	Liu et al. (2024), Yalimaimaiti et al. (2023)

## 5 New directions for synergistic therapy of lung cancer

**FIGURE 4:**
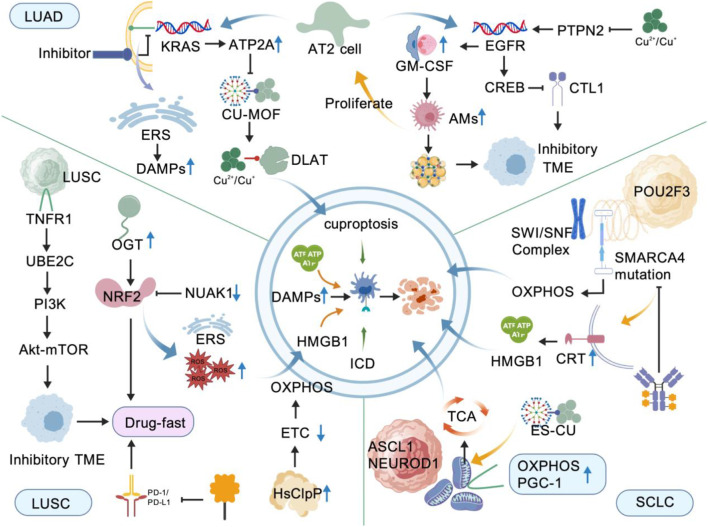
This figure illustrates the subtype-specific precise intervention targets of the cuproptosis-ICD synergistic strategy in the three major lung cancer subtypes. LUAD (Top): KRAS mutation upregulates copper efflux via ATP7A, while Cu-MOF releases Cu^2+^, which binds to DLAT to trigger cuproptosis; KRAS inhibition induces ER stress, which leads to CRT exposure and subsequent ICD; this process releases HMGB1/ATP to enhance CTL infiltration; When EGFR is mutated, Cu^2+^ inhibits PTPN2 to activate CREB; the combination of cuproptosis-ICD and PD-1/L1 blockade reverses immune evasion; ORL or MK1775 synergizes with Cu-MOF to deplete GSH, inhibit FASN, and amplify the cuproptosis-ICD loop. LUSC (Bottom Left): High DLAT expression sensitizes cells to cuproptosis; NUAK1 inhibition downregulates NRF2, which induces ICD via ROS and ER stress; HsClpP activation degrades ETC subunits to inhibit OXPHOS; when combined with PD-1 inhibitors, this strategy reduces Treg and increases CD8^+^ T cells. SCLC (Bottom Right): The ASCL1^+^ subtype is dependent on OXPHOS; Cu^2+^ targets the TCA cycle acyl-modified protein DLAT to induce cuproptosis; The POU2F3^+^ subtype is dependent on SWI/SNF-SMARCA4; its inhibitors not only inhibit tumors but also reduce T cell exhaustion, and can be combined with cuproptosis and ICD to amplify the CAR-T effect.

Studies on cuproptosis and ICDs may provide insights into LUAD, LUSC, and NSCLC, which are the three main types of lung cancer. They exhibit distinct pathological features and molecular mechanisms corresponding to different therapeutic strategies. For example, genetic mutations in *EGFR* and *KRAS* frequently occur in LUAD, but are rare in LUSC. Moreover, KRASG12D can induce the differentiation of Alveolar Type I (AT1) cells into AT2 cells, which further progress to malignancy. Lung cancer usually originates from AT2 cells, and lung cancers derived from AT1 and AT2 cells show opposite responses to Wnt signaling, with the former being more susceptible to inhibition (Juul et al., 2023). LUAD and LUSC can also undergo adenocarcinoma-to-squamous transdifferentiation (AST). Studies have found that the transformation of the two genes is associated with the Wnt pathway; however, there is a critical threshold: if the Wnt pathway is activated beyond this threshold, AST becomes irreversible. In addition, clinical studies have shown that the lower the Wnt level, the higher is the squamous cell component (Fang et al., 2023). This finding suggests that lung cancers with different tissue origins require more precise research and therapeutic regimens. On one hand, in NSCLC, the chemotherapeutic drug pemetrexed (PEM) may induce ICD, activate T cells, and thereby improve the survival period of patients with NSCLC (Sumiyoshi et al., 2021). However, recent studies have revealed that circKIAA1797 inhibits two cuproptosis-related genes, *FDX1* and *LIPT1*, by affecting mitochondria, thereby suppressing cuproptosis and promoting lung cancer progression (Xu H. et al., 2025). This reflects the value of cuproptosis and ICD in lung cancer; however, there is a lack of precise research targeting specific subtypes of lung cancer. In addition, NSCLC and SCLC may transform into one another. This requires us to understand different types of lung cancer and their signature molecular changes, and further explore whether the synergistic therapeutic potential of cuproptosis and ICD in different types of lung cancer can bring new breakthroughs in lung cancer treatment (Figure 4) (Table 2).

**TABLE 2:** Novel directions in synergistic therapy for lung cancer.

Key characteristics/Targets	Lung adenocarcinoma (LUAD)	Lung squamous cell carcinoma (LUSC)	Small cell lung cancer (SCLC)
Major cells of origin	Type II alveolar (AT2) cells (Li Z. et al., 2024)	Bronchial basal cells (Sun et al., 2025)	Neuroendocrine cells (Wang G. Z. et al., 2025)
Key driver mutation	EGFR, KRAS (Aubert et al., 2020; Zhao et al., 2025)	CDKN2A, PI3K/AKT activation (Sun et al., 2024; Satpathy et al., 2021)	ASCL1, NEUROD1, POU2F3 (Wang G. Z. et al., 2025; Varuzhanyan et al., 2024)
Key cuproptosis sensitivity-related molecules	DLAT↑, FDX1↑; KRAS upregulates ATP7A-mediated copper efflux; circKIAA1797 inhibits FDX1/LIPT1 (Xu H. et al., 2025; Zhao et al., 2025)	DLAT↑; FDX1 downregulation activates PI3K/AKT to promote metastasis (Sun et al., 2024); HsClpP regulates ETC (Zhou et al., 2023)	DLAT↑, highly active OXPHOS, SMARCA4 mutation (Solta et al., 2025; Lissanu Deribe et al., 2018)
Mechanism of ICD induction	KRAS inhibition → ER stress → CRT exposure (Nam et al., 2021); EGFR photoimmunotherapy → DAMPs release (Mączyńska et al., 2022); platinum-based chemotherapy induces CRT/ATP/HMGB1 (Furukawa et al., 2021)	NUAK1 inhibition → NRF2↓ → ROS↑ → PERK/eIF2α pathway activation-induced ICD (Gui et al., 2025); Cisplatin + TGF-β inhibitor reverses immunosuppression (Kalfeist et al., 2025)	ICD enhances CAR-T antigen presentation (Li H. et al., 2025); mtDNA leakage activates the cGAS-STING pathway (Tao et al., 2025; Jiang et al., 2025; Liu B. et al., 2025; Li H. et al., 2025)
Metabolic reprogramming characteristics	Lipid metabolism ↑ (GM-CSF/PPARγ) (Kuhlmann-Hogan et al., 2024); FASN dependence (Li Z. Z. et al., 2025); high GSH expression (Kuhlmann-Hogan et al., 2024)	Abnormal fatty acid metabolism; NRF2-mediated antioxidant defense (Kalfeist et al., 2025; Zhang Y. et al., 2024); active glycolysis and PPP (Li Q. et al., 2025)	High OXPHOS dependence; active TCA cycle; high mitochondrial abundance (Solta et al., 2025; Varuzhanyan et al., 2024)
Characteristics of the immune microenvironment	“Cold tumor,” suppressed CD8^+^T cell function (Wang Q. et al., 2025); PD-L1 upregulation (Li Z. Z. et al., 2025; Ross et al., 2024)	Intratumoral Treg/B cell accumulation; strong immunosuppression; decreased CD8^+^T cell infiltration (Zhang H. et al., 2023)	Lack of T cell infiltration; M2-TAM dominance; great potential of CAR-T (Dolkar et al., 2025)
Cuproptosis-ICD synergistic strategy	Cu-MOF + ORL (FASN inhibition) + anti-PD-1 [75]; targeting PTPN2/CREB pathway (Ross et al., 2024); combination with gefitinib or photoimmunotherapy (Mączyńska et al., 2022)	Copper-based nanoplatform + PD-1 inhibitor (Lu et al., 2024); NUAK1 inhibitor + Cisplatin (Gui et al., 2025)	Copper ionophore + SWI/SNF inhibitor + CAR-T (Duplaquet et al., 2024; Battistello et al., 2023; Li H. et al., 2025)

### 5.1 LUAD

LUAD accounts for 7% cancer-related deaths worldwide. It commonly arises in the peripheral lung and is mainly derived from type II alveolar (AT2) cells among the alveolar progenitor cells. These cells can self-renew and differentiate into type I alveolar cells, thereby acting as alveolar stem cells (Li Z. et al., 2024). *KRAS* mutations are found in almost all cancers. Alveolar intermediate cells expressing KRT8 (KRT8-positive alveolar intermediate cells; KACs) are adjacent to human LUAD tissues. *In vitro* studies confirmed that these cells were suppressed by KRAS inhibitors. Interestingly, KACs act as intermediates in the transformation of AT2 cells into cancer cells (Han et al., 2024). Notably, KRAS upregulates ATP7A to prevent the cytotoxicity caused by Cu overload (Aubert et al., 2020). Accordingly, ES-mediated cuproptosis exhibits therapeutic efficacy in *KRAS*-mutant LUAD, which has been validated in mouse models (Zhao et al., 2025). In addition, researchers observed *in vitro* that FDX1-mediated Cu metal–organic frameworks (Cu–MOF) release Cu ions and DLAT in *KRAS*-mutant NSCLC, thereby inducing proteotoxic cell death and achieving effective anti-tumor effects (Li K. et al., 2024), highlighting the cytotoxic effect of Cu overload on tumors. In addition, *KRAS* mutations can alleviate ER stress. Inhibiting KRAS membrane localization in patient-derived cancer cells induces ER stress to “force” cancer cell death, accompanied by a massive DAMP release that trigger ICD and generate more cytotoxic cells against *KRAS* mutations (Nam et al., 2021). Owing to the close association between ICD and autophagy, Cu can also enhance the activity of the autophagy kinase ULK1, which regulates autophagy and drives *KRAS*-mutant LUAD (Tsang et al., 2020). Thus, the synergy between cuproptosis and ICD may yield considerable therapeutic outcomes in LUAD.

In a clinical cohort study by Hill et al. (2023), a high proportion of *KRAS* mutations was detected in normal lung tissues, followed by *EGFR* mutations. When air pollution induces macrophages to infiltrate the lungs and produce interleukin-1β (IL-1β), *EGFR*-mutant AT2 cells undergo stem cell-like changes in their cellular state, thereby promoting LUAD occurrence. Animal experiments further confirmed that *EGFR* mutations drive lung cancer originating from AT2 cells and bronchioalveolar stem cells; however, EGFR inhibitors such as gefitinib are prone to drug resistance (Chen et al., 2021), often accompanied by T cell deficiency. In this case, cell death may provide support: on one hand, high Cu levels can bind to and inhibit the activity of PTPN2, which senses Cu changes and acts as a “brake” for EGFR. This leads to excess activation of EGFR signaling, and phosphorylated EGFR further activates CREB, which has also been demonstrated in LUAD cells. CREB inhibits CTR1, which causes cancer cell resistance to platinum-based drugs (Ross et al., 2024). However, the therapeutic effects of platinum-based drugs in lung cancer may also be mediated by ICD. *In vitro* studies and serological detection in 25 patients showed that chemotherapy-induced apoptosis was positively correlated with cell surface CRT expression for *EGFR*-mutant NSCLC (Furukawa et al., 2021), which may exert anti-tumor effects in immunotherapy. EGFR-targeted photoimmunotherapy can also induce ICD, leading to CRT expression on the tumor cell surface and DAMP release, thereby promoting DC maturation and immune activation; *in vivo* models have shown a reduction in tumor cells (Mączyńska et al., 2022).

In addition, under EGFR oncogenic signaling, AT2 cells secrete large amounts of granulocyte-macrophage colony-stimulating factor (GM-CSF), which in turn enriches alveolar macrophages (AMs) and activates peroxisome proliferator-activated receptor γ (PPARγ) in AMs. This enhances lipid metabolism to support the proliferation of malignant AT2 cells while creating an immunosuppressive microenvironment and upregulating PD-L1 (Kuhlmann-Hogan et al., 2024). However, patient-derived data have shown that abnormal fatty acid metabolism in LUAD is associated with patient survival and may reduce the efficacy of anti-PD-1 therapy. Abnormal lipid metabolism in the TME enhances its immunosuppressive function and inhibits CD8^+^ T cell accumulation. This is further illustrated by the drug MK1775, which targets lipid metabolism and inhibits lipid crosstalk between tumor cells and tumor-associated macrophages (TAMs), thereby enhancing immunotherapy (Chen Y. et al., 2024; Fu et al., 2024). The inhibitory effect of cuproptosis on tumors can also be disrupted by lipid metabolic reprogramming. To address this, researchers have used Cu–MOFs in animal models to release orlistat (ORL) to inhibit fatty acid synthase (FASN), while releasing Cu to induce ROS production and ER alterations, thereby triggering cuproptosis. As a novel form of ICD, cuproptosis upregulates PD-1 (Li Z. Z. et al., 2025), and its combination with anti-PD-1 drugs subsequently exerts a remarkable anti-tumor effect. However, research on therapeutic regimens combining lipid metabolism with ICD and cuproptosis in LUAD remains limited. Nevertheless, cuproptosis combined with ICD plays an important role in the malignant transformation of lung epithelial cells. Further exploration of the associations between LUAD epithelial cell lesions, cutaneous proptosis, and ICD may open new avenues for LUAD treatment.

### 5.2 LUSC

LUSC is the second most common type of lung cancer after LUAD. Among these factors, Cigarette smoking is closely associated with lung squamous cell carcinoma (LUSC). Tobacco exposure downregulates the tumor suppressor Phosphatase and tensin homolog deleted on chromosome 10(PTEN), thereby contributing to tumor progression (Pustylnyak et al., 2023). LUSC usually arises in the upper respiratory tract and central lungs, primarily from basal cells in the pseudo-stratified epithelium with stem cell properties. Abnormal proliferation of these cells can lead to their differentiation into squamous cell carcinoma, which subsequently progresses to undifferentiated spindle cell carcinoma. Owing to the absence of targetable mutations in LUSC, the only current therapeutic options are chemotherapy and immune checkpoint inhibitors; however, drug resistance remains a major challenge in chemotherapy. *In vitro* and *in vivo* models have shown that transforming growth factor-β (TGF-β) inhibitors can reverse drug resistance in LUSC, which may involve the activation of epithelial–mesenchymal transition (EMT) enriched in tumor cells (Sun et al., 2025). TGF-β promotes EMT through canonical and non-canonical pathways, transforming epithelial cells into mesenchymal-like cells to facilitate cancer cell invasion and metastasis. TGF-β also interacts with matrix metalloproteinases (MMPs) to exert its biological activities. Owing to the severe adverse effects of TGF-β inhibitors, Cu, often serving as a cofactor for metalloenzymes, can accelerate lung cancer progression, while Cu chelators can downregulate MMPs to reduce TGF-β levels (Poursani et al., 2023). Increased DLAT levels reduce its acylation, preventing it from binding to Cu ions and thereby downregulating Cu synthesis. In hepatocellular carcinoma cells, high expressions of DLAT, glutaminase (GLS), and cyclin-dependent kinase inhibitor 2A (CDKN2A) are associated with TGF-β activation (Wang et al., 2024b). The chemotherapeutic drug cisplatin can also stimulate cells to produce TGF-β, which in turn induces the accumulation of immunosuppressive cells such as regulatory T (Treg) cells. In this context, inhibiting TGF-β can restore the function of CD8^+^ T cells, triggering cisplatin-induced ICD and thereby exerting an anti-tumor effect (Kalfeist et al., 2025). However, when cisplatin is administered to lung cancer tissues, O-GlcNAc transferase (OGT) increases glycosylation of the antioxidant molecule nuclear factor erythroid 2-related factor 2 (NRF2), thereby promoting NRF2 stabilization. NRF2 promotes cisplatin resistance in cancer cells (Zhang Y. et al., 2024). Meanwhile, the immune checkpoint inhibitor anti-PD-L1 tiragolumab combined with chemotherapy serves as a first-line treatment regimen for advanced LUSC. A retrospective study of patients with LUSC revealed that NRF2 expressed in LUSC can directly upregulate PD-L1; however, this PD-L1 upregulation is not indicative of abundant IFN-γ-producing T cells around the tumor, thereby leading to resistance to anti-PD-L1 therapy and making it difficult to accurately predict its therapeutic efficacy (Duan et al., 2023). Serine/threonine kinase 1 (NUAK1) is frequently expressed in various types of cancers and upregulates NRF2 to protect cancer cells. When NUAK1 is inhibited, researchers can observe a subsequent decrease in NRF2, which induces ROS production to promote ER stress and trigger ICD, thereby exerting an anti-tumor effect (Gui et al., 2025). Furthermore, cuproptosis-inducing nanoplatforms can amplify immune responses, reduce the number of immunosuppressive cells, and exhibit favorable anti-tumor effects in animal experiments when combined with PD-1 inhibitors (Lu et al., 2024). Therefore, in the future, the application of cuproptosis and ICD in LUSC tissues may yield remarkable anti-tumor outcomes.

The most aggressive undifferentiated spindle cell carcinoma represents the terminal stage of LUSC and exhibits EMT characteristics. Tumor necrosis factor receptor 1 (TNFR1) can promote the transformation of well-differentiated squamous cell carcinoma into undifferentiated spindle cell carcinoma. In this context, UBCH10 serves as an effector protein target downstream of TNFR1 (Xiao et al., 2022). UBE2C, which encodes the UBCH10 protein, activates the Akt–mTOR pathway. The PI3K–Akt–mTOR pathway is a fundamental signaling pathway that promotes cell growth, survival, metabolism, and proliferation, and its aberrant activation frequently occurs in various cancers (Li Q. et al., 2022; Lv et al., 2023). Moreover, in LUSC chemoradiotherapy, the Akt–mTOR pathway is the most active signaling pathway in drug-resistant tumors (Shen et al., 2022). FDX1 downregulation activates the PI3K/AKT pathway and mitophagy to promote cancer cell metastasis. Mitophagy (mitochondrial autophagy) can eliminate damaged mitochondria caused by excess tumor cell growth, whereas FDX1 promotes mitophagy by stabilizing mitochondria and reducing ROS (Sun et al., 2024). Direct Myc activation upregulates mTORC1; at this point, mTOR inhibitor use leads to eIF4E sequestration, unfolded protein accumulation in the ER, and PERK–eIF2α–CHOP pathway initiation to promote ER stress, which in turn triggers ICD to exert an anti-tumor effect (Leibowitz et al., 2023). CDKN2A acts as a “brake” for CDK4/cyclin-D1; when CDKN2A is inactivated, CDK4/cyclin-D1 accelerates LUSC progression. However, when *CDKN2A* is mutated in LUSC, its mRNA expression increases (Satpathy et al., 2021). In contrast, in hepatocellular carcinoma, CDKN2A reduces Cu ions by regulating Cu-related transporters, thereby activating the Wnt pathway and inhibiting cuproptosis (Cheng et al., 2024). Precancerous lesions of LUSC are also frequently accompanied by early CDK4/cyclin-D1 activation and late PI3K/Akt pathway activation. Furthermore, the progression of endobronchial precancerous lesions into progressive squamous cell carcinoma is likely driven by immune escape (Roberts et al., 2023). Unlike the immune escape mechanism in LUAD, more lymphocytes are recruited into the tumor interior in LUSC than in the peritumoral area, which is associated with the high mortality rate of LUSC, but has no impact on the efficacy of immunotherapy in LUAD. At this point, many intratumoral B cells accumulate and form an immunosuppressive microenvironment together with a high proportion of Tregs, leading to a reduction in CD8^+^ T cells (Zhang H. et al., 2023). Mitochondrial caseinolytic protease P (HsClpP) is a serine protease that degrades defective mitochondrial proteomes. Since most mitochondrial proteins serve as “carriers” for ATP production via the electron transport chain (ETC), Zhou et al. (Zhou et al., 2023) targeted and activated HsClpP to degrade ETC subunits, thereby inhibiting mitochondrial OXPHOS. This facilitates cell growth arrest in LUSC and exerts an anti-tumor effect. The “fuel” for the ETC is derived from the TCA cycle. DLAT and DLST not only regulate carbon entry into the TCA cycle, but also bind to Cu ions to induce cuproptosis (Moison et al., 2024). Therefore, further exploration of the characteristic molecular changes in LUSC and their alterations under the action of ICD and cuproptosis may reveal new therapeutic targets.

### 5.3 SCLC

SCLC originates from neuroendocrine (NE) cells and is the most malignant and invasive lung cancer. Owing to the lack of surgical opportunities in most cases, combined chemotherapy regimens are typically adopted for treatment. SCLC can be classified into NE and non-NE types. In a cohort study, Wang et al. used single-cell sequencing and found that 74.38% SCLC cells highly express the NE transcription factor ASCL1 (Wang G. Z. et al., 2025). ASCL1 promotes NE cell proliferation in SCLC. Studies have shown a characteristic increase in OXPHOS in ASCL1-subtype cells, accompanied by NADH dehydrogenase enrichment and increased mitochondria (Solta et al., 2025). Consistently, OXPHOS has been identified in small cell NE (SCN) carcinomas from various tissue origins, accompanied by high expression of peroxisome proliferator-activated receptor γ coactivator 1α (PGC-1α). In human prostate tissues, ASCL1-subtype SCN carcinomas exhibit high OXPHOS activity (Varuzhanyan et al., 2024). This further indicated a possible close association between ASCL1-subtype SCLC and OXPHOS. Cuproptosis specifically targets mitochondrial respiration and acylates proteins in the TCA cycle, thereby triggering proteotoxic stress. Thus, Cu exhibits high sensitivity in hepatocellular carcinoma cells, which are highly dependent on mitochondrial respiration (Xing et al., 2023). This study also provides insight into Cu-based therapies for ASCL1-subtype SCLC. Furthermore, SMARCA4, an ATPase catalytic subunit of the SWI/SNF complex, exerts distinct effects on SCLC subtypes with low- and high-NE features. In two NE-type SCLCs (ASCL1 and NEUROD1), SMARCA4 contributes to the maintenance of high-NE characteristics (Redin et al., 2024). In contrast, the SWI/SNF complex plays a “pyramid-like” pivotal role in the non-NE POU2F3-positive SCLC subtype. Recent studies have confirmed that the SWI/SNF complex mediates *POU2F3* regulation in a targeted manner, and inhibitors targeting SMARCA4 (the ATPase catalytic subunit of this complex) inhibit SCLC; however, the reduction of tumors remains to be addressed (Duplaquet et al., 2024). In this context, combination therapy may provide new hope. On one hand, as a tumor suppressor gene, *SMARCA4* exhibits high cellular respiration and OXPHOS activity after mutation in lung cancer; additionally, lung cancer with *SMARCA4* mutation shows certain sensitivity to OXPHOS inhibition. Based on this, we propose that combining cuproptosis (which targets cellular respiration) may facilitate SCLC regression (Lissanu Deribe et al., 2018). In contrast, experiments using human- and mouse-derived cells have shown that SWI/SNF complex inhibition is beneficial for enhancing the presence of T cells *in vitro* and *in vivo*, delaying T cell exhaustion, and thereby creating favorable conditions for CAR-T cells (Battistello et al., 2023). CAR-T cells targeting human SCLC are currently undergoing clinical trials (Dolkar et al., 2025). CAR-T cells are a therapeutic approach in which isolated T cells are genetically modified and re-infused into the body to kill cancer cells by targeting specific tumor antigens. Li et al. reported that stimulating ICD in tumor cells facilitates tumor-associated antigen presentation to CAR-T cells by DCs (Li H. et al., 2025). Combined with the intrinsic ability of ICD to trigger adaptive immune responses, this strategy markedly reduced solid tumor volume. In summary, we believe that mitochondrial respiration involved in cuproptosis, as well as the role of ICD in SCLC should be further investigated.

We have previously described the feasibility and molecular mechanisms underlying the synergistic therapy of lung cancer using cuproptosis and ICD. However, the clinical translation of this strategy relies critically on establishing actionable biomarkers for patient stratification. According to current evidence, three progressive biomarker strategies can be defined. First, subtype-specific driver molecules, such as *KRAS*/*EGFR* mutations in LUAD, *CDKN2A* mutations in LUSC, and high ASCL1 expression and *SMARCA4* mutations in SCLC, are critically important for subtype classification and risk stratification (Mao et al., 2025). Among functional indicators of cuproptosis, relative exchangeable Cu (REC) serves as an important biomarker for Cu metabolism, which can dynamically reflect the tolerance threshold of the organism to cuproptosis inducers (Djebrani-Oussedik et al., 2025). Furthermore, immune activation imaging biomarkers, such as the Cu-PET/CT molecular probe, enable the visualization of immune cell activity, inflammatory status, and immunotherapy response, thereby providing pharmacodynamic feedback for the synergistic therapy of cuproptosis combined with ICD (Tao et al., 2025). Stratified biomarker studies can prospectively identify beneficial populations, reduce systemic toxicity, and provide effective screening criteria for clinical trials (Table 3).

**TABLE 3:** Summary table of key studies.

Model	Intervention study	Monitoring indicators	Merits and drawbacks	Correlation with lung cancer	References
Animal model	Induction of cuproptosis by elesclomol	Tumor regression degree	To date, there is no direct evidence for a therapeutic role of ICD in KRAS-mutant LUAD. Nevertheless, it can still provide a research background for KRAS-mutant tumors	Provide a reference for the treatment of KRAS-mutant LUAD by exploiting cuproptosis and ICD	Zhao et al. (2025)
Patient-derived model	Inhibition of KRAS membrane localization	DAMPs			Nam et al. (2021)
*In vitro* model	High copper	EGFR	To date, studies have remained at the preclinical stage, and direct evidence linking cuproptosis to EGFR is lacking. Nevertheless, these findings still provide a research framework for EGFR-mutant tumors	Provide a reference for the treatment of EGFR-mutant LUAD by exploiting cuproptosis and ICD.	Ross et al. (2024)
Animal model	EGFR-targeted photoimmunotherapy	DAMPs and DC			Mączyńska et al. (2022)
Patient-derived models, *in vitro* models, and animal models	ORL@Cu-MOF-triggered cuproptosis and suppression of fatty acid metabolism	Correlation between DLAT and FASN expression, CD4/CD8+ T cells, and tumor size	The relationship between DLAT and FASN has been elucidated in cancer cells; however, direct evidence is still lacking in lung adenocarcinoma. Nevertheless, it provides a certain reference for the study of lung adenocarcinoma	Dysregulated lipid metabolism impairs the therapeutic efficacy of lung adenocarcinoma, in which overexpression of FASN reduces the infiltration of CD8^+^ T cells in lung adenocarcinoma. Furthermore, other studies have suggested that DLAT may be positively correlated with the expression of fatty acid synthase (FASN)	Li Z. Z. et al. (2025)
*In vitro* and *in vivo* animal models	MK-1775, a sensitizer targeting lipid metabolism, was used in this study	PD-L1, tumor size, CD8^+^ T cells			Chen Y. et al. (2024)
*In vitro* and *in vivo* models	TGF-β inhibitor	The sensitivity of tumor cells to chemotherapeutic drugs	These studies are still limited to the preclinical stage. The direct evidence of TGF-β in LUSC, as well as the potential crosstalk among TEPA, ICD, and TGF-β, may provide novel research directions for LUSC.	TGF-β inhibitors can reverse drug resistance in lung squamous cell carcinoma (LUSC). Studies have shown that TGF-β signaling blocks chemotherapy-induced immunogenic cell death (ICD), thereby conferring drug resistance, whereas copper chelators help reduce TGF-β levels	Sun et al. (2025)
Animal model	Copper chelator TEPA	TGF-β, EMT markers			Poursani et al. (2023)
Animal model	Combination of CDDP, Eri, and anti-TGF-β antibody	Temporal analysis of animal survival rate, TGF-β, and immunosuppressive cell populations			Kalfeist et al. (2025)
*In vitro* experiments and patient-derived models	Proteomic analysis and ToppCluster enrichment	Oxidative phosphorylation levels	To date, direct evidence targeting cuproptosis and immunogenic cell death (ICD) in small cell lung cancer (SCLC) remains lacking. However, multiple studies have suggested that targeting the characteristic alterations of SCLC via the combination of these two forms of cell death may yield favorable therapeutic efficacy	Small cell lung cancer (SCLC) is characterized by elevated oxidative phosphorylation (OXPHOS) levels, and cuproptosis targets cellular respiration precisely. Meanwhile, inhibition of the SWI/SNF complex suppresses tumor growth and delays T-cell exhaustion. Based on these findings, CAR-T therapy targeting SCLC is currently under clinical trials, and immunogenic cell death (ICD) enhances the recognition of antigens presented by DCs for CAR-T cells, providing a combinatorial strategy for SCLC treatment	Solta et al. (2025)
*In vitro* experiments	FHD-286, a small-molecule inhibitor of the mSWI/SNF ATPase subunits	Cell proliferation assay, POU2F3			Duplaquet et al. (2024)
*In vitro* experiments	mSWI/SNF-targeted intervention	T cell function, survival indicators, and T cell memory differentiation lineage			Battistello et al. (2023)
*In vitro* experiments and animal models	Triboelectric Immunotherapy	Cell viability, ATP and HMGB1 levels, and DCs			Li H. et al. (2025)

## 6 Challenges and pathways for clinical translation

Although the synergistic strategy of cuproptosis and ICD has shown great potential in preclinical models, a series of translational medicine challenges, including biomarker screening, treatment safety, and administration strategies, must be overcome before it can be translated into a mature clinical therapeutic regimen. Based on existing evidence, constructing a complete translational pathway is key to achieving this goal.

### 6.1 Biomarkers and patient stratification

Precise patient stratification is a prerequisite to ensure therapeutic efficacy. Owing to the high heterogeneity of lung cancer, we propose a stratification based on a multi-dimensional biomarker system. Upstream stratification is performed according to the genetic background of the tumor, which determines the fundamental metabolic characteristics of lung cancer. Patients with KRAS-mutant LUAD often exhibit a high demand for Cu metabolism and maintain Cu homeostasis via ATP7A upregulation (Zhao et al., 2025; Aubert et al., 2020), thus representing a potentially eligible population for Cu ionophore therapy. In contrast, patients with SCLC harboring SMARCA4 deficiency or high ASCL1 expression heavily rely on mitochondrial respiration (Lissanu Deribe et al., 2018) and are sensitive to cuproptosis inducers targeting the TCA cycle. Subsequently, stratification was performed at the intermediate level according to the sensitivity markers of cuproptosis. FDX1 and protein lipoylation levels are the core indicators of therapeutic efficacy. Tsvetkov et al. have clearly demonstrated that FDX1 abundance is positively correlated with the sensitivity of various tumor cells to cuproptosis inducers (Shi Z. et al., 2025; Tsvetkov et al., 2022; Wang X. et al., 2023). Therefore, detecting FDX1, DLAT, and LIPT1 expression levels in tumor tissues by immunohistochemistry (IHC) can screen the “cuproptosis-sensitive” subgroup. In addition, REC, a highly specific indicator reflecting body Cu load, can be used to evaluate baseline Cu metabolism in patients (Guo et al., 2025). Finally, downstream stratification was performed based on the monitoring of immunity and ICD effects, and monitoring after treatment should focus on ICD-related signals. Dynamic changes in serum HMGB1 protein, HSP70 in exosomes, and ATP levels can serve as “liquid biopsy” indicators for determining successful ICD induction (Fan et al., 2024). Meanwhile, using the 64Cu-PET/CT molecular imaging probe, Cu accumulation at the tumor site and the subsequent immune cell infiltration status can be visualized real-time, providing intuitive evidence for therapeutic efficacy evaluation (Tao et al., 2025). By establishing a multi-dimensional “stratification-prediction-monitoring” biomarker system, we aim to provide more precise medical plans for patients with lung cancer.

### 6.2 Therapeutic feasibility and delivery systems

Another core challenge in clinical translation lies in balancing therapeutic efficacy and systemic toxicity as well as achieving precise delivery. Constructing intelligent delivery systems using nanotechnology is a key and feasible approach to solving this problem. At present, responsive release carriers designed by utilizing the TME characteristics (high GSH and low pH value) have been developed, such as Cu–MOF (Li Z. Z. et al., 2025), ES–Cu–MOF nanofactors (Luo et al., 2024), and Elesclomol–Cu@PDPA nanoparticles (PEC NPs) (Yun et al., 2025). These carriers are stable in the blood circulation and only release Cu ions and inducers at the tumor site, thereby significantly improving tumor selectivity. Cuproptosis exhibits significant dose dependence and a strict “therapeutic window” must be established. Specifically, it is necessary to not only reach the threshold for triggering FDX1-mediated Cu synthesis in tumor cells, but also control the serum Cu level within the hepatic compensatory capacity of ATP7B/ATOX1 to avoid severe hepatorenal toxicity (Mao et al., 2025). In addition, the timing sequence of the combination medication is crucial. A “sensitization first, then attack” strategy should be adopted: specifically, cuproptosis inducers are first used to trigger ICD and reshape the immune microenvironment; after the “cold tumors” turn hot, immune checkpoint inhibitors (ICIs) or CAR-T therapy are then administered to maximize the synergistic effect.

### 6.3 Safety and toxicity management

Safety considerations are represented by red lines in the clinical trial design. The liver is a central organ involved in systemic Cu metabolism. The Cu-transporting P-type ATPase ATP7B is predominantly expressed in hepatocytes and responsible for transporting excess intracellular Cu into the bile for excretion, thereby maintaining systemic Cu homeostasis (Lutsenko et al., 2007). Therefore, during the clinical application of cuproptosis inducers, liver function indicators [alanine aminotransferase (ALT)/aspartate aminotransferase (AST)], ceruloplasmin, and 24-h urinary Cu should be regularly monitored to evaluate the hepatic Cu excretion function and potential accumulation risks. Long-term Cu overload may induce neurotoxicity similar to Wilson’s disease, requiring neurological evaluation every 3 months (Tsvetkov et al., 2022). Cuproptosis relies on an active TCA cycle. Normal tissues have low OXPHOS levels, which theoretically possesses a therapeutic window (Tsvetkov et al., 2022). However, attention should still be paid to the particularities of lung tissue, such as potential damage caused by high blood flow and high oxygen levels. Cu-chelating “rescue agents,” such as penicillamine derivatives, can be used for emergency intervention in cases of severe toxicity. The strong inflammatory response induced by cuproptosis and the large number of cytokines released during ICD carry the risk of inducing cytokine release syndrome (CRS) or exacerbating autoimmune pneumonia and myocarditis when combined with anti-PD-1/L1 therapy. This requires close monitoring of inflammatory factors such as IL-6 and TNF-α in clinical trials, as well as the formulation of corresponding immunosuppressive plans (Xu Y. et al., 2025; Ji et al., 2025).

### 6.4 Clinical positioning

Based on the existing preclinical and early clinical evidence, the potential clinical application scenarios of the cuproptosis–ICD synergistic therapy mainly cover the following three dimensions: (I) Drug Resistance Reversal Strategy: For patients with epidermal growth factor receptor–tyrosine kinase inhibitor (EGFR–TKI)-resistant LUAD or chemotherapy-resistant LUSC, the unique mechanism of the cuproptosis-independent apoptotic pathway is utilized to overcome classical therapeutic resistance caused by Bax/Bak deficiency or Caspase inactivation (Wang X. et al., 2023), providing an alternative intervention for later-line therapy. (II) Immunotherapy Sensitization Strategy: For SCLC and LUAD with low PD-L1 expression or an immune-desert phenotype, ICD induction is used to reshape the tumor immune microenvironment. As a sequential or combined regimen, it enhances the response rate to immune checkpoint inhibitors and achieves phenotypic conversion of “cold tumors” to “hot tumors.” (III) Efficacy Evaluation Endpoints: In short term, the ORR was used as the primary observation indicator, while the long-term focus was on prolonging progression-free survival (PFS). It is particularly suitable for patients with advanced lung cancer for whom existing standard treatments have failed, aiming to meet unmet clinical needs in this field.

In summary, the synergistic strategy of cuproptosis and ICD has opened a new paradigm for the precise treatment of lung cancer. The construction of its translational pathway relies on a three-in-one clinical framework of “multi-dimensional biomarker stratification - intelligent nano-delivery system - full-course toxicity monitoring.” In the future, with the standardization of FDX1/protein lipoylation detection, iterative optimization of TME-responsive nanocarriers, and maturation of dynamic imaging monitoring technology for Cu metabolism, this strategy is expected to overcome existing therapeutic bottlenecks. This will provide a closed-loop solution from “biomarker-guided precise stratification” to “individualized sequential combination medication” for patients with drug-resistant and immunologically cold tumors, ultimately leading to a leap from a preclinical concept to a standard treatment option for lung cancer.

## 7 Summary and outlook

The synergistic effects of cuproptosis and ICD provide a novel intervention strategy with mechanistic feasibility, classification accessibility, and marker detectability for lung cancer treatment. Combined therapeutic regimens are commonly adopted in the current treatment of lung cancer; however, owing to issues such as drug resistance, tumor lineage plasticity, and systemic Cu homeostasis, there is an urgent need for new combined therapeutic regimens. Moreover, current research still faces limitations. For example, an immunosuppressive state in the tumor microenvironment of LUAD is characterized by inhibition of CD8^+^ T cell function mediated by lactic acid secreted by cancer-associated fibroblasts. This mechanism severely impairs ICD-induced anti-tumor immune response, which illustrates the importance of combined medication or therapy (Wang Q. et al., 2025). Therefore, future research should explore combined intervention strategies, such as simultaneously targeting lactic acid metabolism and cuproptosis pathway, to reverse the immunosuppressive microenvironment and achieve conversion from “cold tumors” to “hot tumors.” In addition, almost all existing evidence is derived from cell models or mouse experiments, and various cuproptosis inducers (such as elesclomol–Cu, Cu–MOF, and ORL@Cu–MOF) have shown good anti-tumor and immune activation effects in animals. However, their safety, pharmacokinetic characteristics, and clinical efficacy in humans have not yet been verified. The distribution, accumulation, and potential toxicity of Cu ions in the body require systematic evaluation. Promoting the translation of the cuproptosis–ICD synergistic strategy from preclinical research to early clinical trials is a key step toward realizing the clinical value of this theory.

In summary, the molecular interaction network between cuproptosis and ICD in different types of lung cancer should be analyzed. Moreover, understanding the three core propositions of Cu homeostasis toxicity prevention and control, stratification marker verification, and translational delivery systems, as well as developing implementable combined therapeutic regimens based on the cuprotosis–ICD interplay will be an important direction for precise immunotherapy of lung cancer.
